# Special Issue: “Inflammation, Oxidative Stress and Protein Aggregation; Any Links?”

**DOI:** 10.3390/cells9112461

**Published:** 2020-11-11

**Authors:** Eva Žerovnik, Salvador Ventura, Nataša Kopitar Jerala

**Affiliations:** 1 Department of Biochemistry and Molecular and Structural Biology, Jožef Stefan Institute, 1000 Ljubljana, Slovenia; natasa.kopitar@ijs.si; 2 Institute of Biotechnology and Biomedicine & Department of Biochemistry and Molecular Biology, Autonomous University of Barcelona, 08193 Bellaterra, Spain; salvador.ventura@uab.es

Inflammation is a complex immune response that enables survival during infection and maintains tissue homeostasis. In response to pathogens, damaged cells, or irritants a cascade of signals leads to the recruitment of immune cells. Sustained robust inflammation may lead to serious disorders due to the overproduction of pro-inflammatory cytokines and tissue damage. 

Proteostasis (proportion between the synthesis, translation, folding, and degradation of proteins) should be in balance in a healthy cell, including neurons. However, due to different stressors among them, such as heat (fever, infection), acidification (lack or excess of nutrients), and oxidative stress, the mis-folding and aggregation of otherwise normal sequences of proteins occurs. Oxidative stress increases upon protein aggregation, due to metals binding to the aggregates, membrane perturbation, or permeabilization by soluble aggregates.

In this Special Issue, papers have been collected in which authors discuss how oxidative stress influences the inflammatory immune response: which external factors influence protein aggregation, and how neuro-regeneration can be restored by dietary means. 

In the first paper by Santos et al., Ventura, the authors describe an empirical equation to model the pH-dependent aggregation of intrinsically disordered proteins (IDPs) [1]. Their assumption is that both the global protein charge and lipophilicity depend on the solution pH. This simple phenomenological approach showed unprecedented accuracy in predicting pH dependence of the aggregation of both pathogenic and functional amyloidogenic IDPs.

In the second paper by Trstenjak-Prebanda et al., the authors examined the role of cysteine proteinase inhibitor—stefin B (cystatin B) in oxidative stress [2]. Specifically, the authors investigated how does Lipopolysaccharide (LPS)-induced oxidative stress affected the protein levels and redox status of redox sensitive proteins—thioredoxin, peroxiredoxins, and superoxide dismutases in macrophages and spleens of LPS-injected mice. LPS challenge was found to result in a marked elevation in mitochondrial peroxiredoxin 3, sulfiredoxin, and superoxide dismutase 2 in stefin B-deficient macrophages and spleens. In addition, they determined that sulfiredoxin was targeted to mitochondria after LPS challenge. They concluded that the upregulation of mitochondrial redox-sensitive proteins, peroxiredoxin 3 and mitochondrial superoxide dismutase 2 in stefin B-deficient cells, implies a protective role of stefin B in mitochondrial function.

In the third paper by Carija et al., Ventura, the authors explore how proteins’ 3D structures evolved to minimize the risk of aggregation in natural environments [3]. By exploiting the AGGRESCAN3D structure-based approach to predict the aggregation propensity of >600 *Escherichia coli* proteins, they show that the structural aggregation propensity of globular proteins is connected with their abundance, length, essentiality, subcellular location, and quaternary structure. The proteome-wide analysis thus suggests that prevention of aggregation upon misfolding plays a key role in sequence evolution and that the avoidance of protein aggregation has contributed to shape the structural properties of proteins. Figure 1 shows different scenarios of Gibbs free energy towards energy of activation, whose ratio determines the relative propensity to aggregation.

In the fourth review paper, by Pogačnik, Ota and Poklar-Ulrih, the authors explore what clinical studies tell about the possible positive effect of lifelong consumption of dietary anti-oxidant substances, such as phytochemicals (e.g., polyphenols) and endogenous substances (e.g., acetyl-l-carnitine, coenzyme Q10, n-3 polysaturated fatty acids) [4]. Can such a regime postpone protein aggregation in neurodegenerative diseases? They stress the importance of availability of these substances to the central nervous system, where they have to be present in high enough concentrations in order to exhibit their neuroprotective properties. Their aim was to summarize their mechanisms of action and therapeutic considerations, including bioavailability and safety data. 

## Figures and Tables

**Figure 1:**
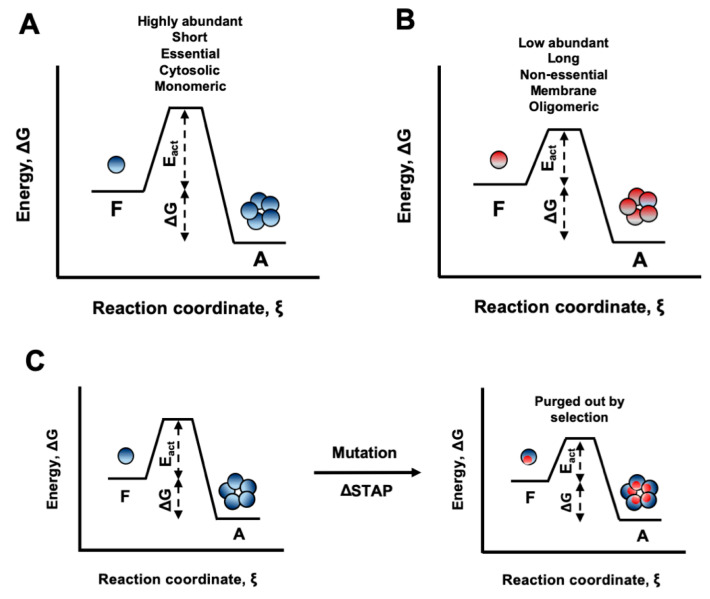
Schematic representation of the energy diagrams for aggregation processes. The ensemble of folded proteins in solution is considered as one thermodynamic system. The aggregated state represents in all cases the global minimum of Gibbs energy. Activation energies (E_act_) are a barrier that determine the rate of transition. (**A**) Proteins with low STAP, such as highly abundant, short, and essential ones and proteins active as monomers, are protected from aggregation by a high energy barrier. (**B**) Proteins with higher STAP, such as low abundant, long and non-essential ones and proteins active in oligomeric forms, have to cross a lower energy barrier. (**C**) Mutations that increase the STAP without conferring additional functional advantages would be purged out by natural selection, since they decrease the energy barrier for aggregation from the initially folded state. STAP = structural aggregation propensity. Figure is taken from the third paper [3].
